# UAV Path Optimization for Angle-Only Self-Localization and Target Tracking Based on the Bayesian Fisher Information Matrix

**DOI:** 10.3390/s24103120

**Published:** 2024-05-14

**Authors:** Kutluyil Dogancay, Hatem Hmam

**Affiliations:** 1 UniSA STEM, University of South Australia, Mawson Lakes Campus, Mawson Lakes, SA 5095, Australia; 2 Defence Science & Technology Group, Sensors and Effectors Division, Edinburgh, SA 5111, Australia; hatem.hmam@defence.gov.au

**Keywords:** self-localization, target tracking, autonomous vehicles, Bayesian Fisher information matrix, A-optimality criterion, D-optimality criterion, Kalman filter

## Abstract

In this paper, new path optimization algorithms are developed for uncrewed aerial vehicle (UAV) self-localization and target tracking, exploiting beacon (landmark) bearings and angle-of-arrival (AOA) measurements from a manoeuvring target. To account for time-varying rotations in the local UAV coordinates with respect to the global Cartesian coordinate system, the unknown orientation angle of the UAV is also estimated jointly with its location from the beacon bearings. This is critically important, as orientation errors can significantly degrade the self-localization performance. The joint self-localization and target tracking problem is formulated as a Kalman filtering problem with an augmented state vector that includes all the unknown parameters and a measurement vector of beacon bearings and target AOA measurements. This formulation encompasses applications where Global Navigation Satellite System (GNSS)-based self-localization is not available or reliable, and only beacons or landmarks can be utilized for UAV self-localization. An optimal UAV path is determined from the optimization of the Bayesian Fisher information matrix by means of A- and D-optimality criteria. The performance of this approach at different measurement noise levels is investigated. A modified closed-form projection algorithm based on a previous work is also proposed to achieve optimal UAV paths. The performance of the developed UAV path optimization algorithms is demonstrated with extensive simulation examples.

## 1. Introduction

Target tracking using AOA measurements has been studied extensively. A critical assumption made when collecting AOA measurements is that the sensor location is known either exactly or is subject to some uncertainty or noise. A GNSS receiver is commonly used to determine the sensor location in outdoor environments. In situations where the GNSS is either not available or cannot be relied upon, beacons or landmarks can provide an alternative means for sensor self-localization using, for example, beacon bearings obtained from an imaging system.

Self-localization using beacons or landmarks is an active research area, especially in autonomous robotics. Early work focused on developing low-complexity linear estimators for the position and orientation of a robot from landmark bearings [1]. A two-stage algorithm consisting of a coarse search followed by a closed-form solution was proposed in [2] using bearing measurements from three beacons or landmarks. Improved pseudolinear estimators for self-localization from landmark bearings were developed in [3,4]. In [5], the vision-based self-localization methods used in the RoboCup middle-size league competition were reviewed and their performances were assessed in a robot soccer environment. A self-localization approach for a mobile robot using multiple candidate landmarks and an omni-directional vision system was proposed in [6]. A range-only simultaneous localization and mapping (SLAM) algorithm was developed in [7] using range measurements acquired from a sonar sensor and inter-node distance measurements for networked landmarks. This approach does not use direction-of-arrival information from landmarks, therefore making it well suited for environments where a clear line of sight to landmarks is not available. In [8], hop-count-distance-based self-localization was considered in a wireless network and the effect of landmark placement on the self-localization accuracy was studied. An improved robot self-localization method was proposed in [9] by estimating robot odometric errors and landmark poses independently from the robot pose, thereby allowing the use of dynamic landmarks in addition to fixed landmarks. Distributed sensor self-localization algorithms were developed in [10] for networked beacons where each node is able to broadcast information about its own position to facilitate self-localization for all nodes. In [11], a low-cost self-localization method suitable for indoor robots was developed using wheel-based odometry, bearing measurements from acoustic beacons, and prediction of beacon bearings from self-localization and orientation estimates. In [12], the performance of a beacon-based self-localization system was studied and a neural network approach was proposed for making design decisions in relation to optimal beacon placement and inference of robot self-localization from noisy measurements in a given environment. Vehicle pose estimation (position and orientation) from multi-modal sensor information and a reference map that contains the landmarks within the vehicle’s field of view were considered in [13]. A deep neural network was developed to learn the mapping between the measured landmarks and the reference map.

Sensor path optimization to maximize the accuracy of self-localization is a research topic of significant interest. UAV trajectory optimization for self-localization using 3D landmarks was studied in [14]. A 3D random sample consensus algorithm was developed, incorporating a modified Kalman filter. In [15], an indoor self-localization algorithm for a swarm of robots was proposed using active optical beacons. Optimal UAV trajectories for stationary and mobile beacons were investigated in [16] using the covariance of predicted Kalman filter state estimates [17] and building on the D-optimality criterion [18]. Self-localization methods for UAVs commonly use optical sensors for landmark or beacon angle measurements with respect to the UAV location and orientation. A comprehensive review of visual and optical sensing methods for self-localization is available in [19].

Once the sensor locations are estimated, the target AOA measurements can be processed to produce target tracks. Finding optimal UAV trajectories for AOA target localization and tracking has been researched extensively (see, e.g., [20] and the references therein). In this paper, our objective is to optimize the flight path of a single UAV, equipped with an AOA sensor collecting bearing measurements from beacons and a manoeuvring target, for joint self-localization (UAV location and orientation estimation) and target tracking. The joint estimation problem is solved optimally by modelling the interactions between the UAV location estimates and target track estimates in a recursive Bayesian estimation framework. It is worth mentioning that treating self-localization and target tracking separately as a two-stage estimation problem, where the self-localization errors are not fully accounted for by the target tracker, would yield non-optimal results. Despite its immense potential for practical application, the problem of UAV path optimization for joint self-localization and target tracking has not received much attention in the literature. In [21], a Kalman filtering approach for self-localization and target tracking was proposed under the strong assumption of perfect knowledge of UAV orientation, which is not justified in practice. The optimization criterion adopted in [21] minimizes the trace of the covariance of Kalman filter state estimates, akin to [17,18]. However, the entire state vector (including the velocity estimates) of the Kalman filter, rather than only target location estimates, is used in the optimization, which does not prioritize target tracking and increases the computational complexity as a result of increased matrix dimensions.

In this paper, new UAV path optimization algorithms are developed for the joint estimation problem of UAV self-localization and target tracking. The estimation problem is first cast into a Kalman filtering framework in the 2D plane using an augmented state vector which includes the target and UAV kinematic parameters (location and velocity vectors) and the UAV orientation angle. This formulation then allows the recursive Bayesian Fisher information matrix (BFIM) [22] to be adopted as a performance bound, as well as the basis for determining optimal UAV paths in a principled manner. Scalar measures of optimization are obtained from A- and D-optimality criteria which minimize the trace of the inverse of the recursive BFIM and maximize the determinant of the recursive BFIM, respectively [23,24,25]. The main contributions of the paper are as follows:

- An augmented Kalman filter formulation for joint angle-only self-localization (including orientation estimation) and target tracking, which provides an optimal and convenient solution to deal with self-localization and orientation uncertainties, and their impact on target tracking performance.
- A- and D-optimality criteria for UAV path optimization using an approximate BFIM and focusing on target location estimates in the augmented Kalman filter state vector for accurate optimization results in the face of self-localization uncertainties.
- Analysis of measurement noise effects on optimal UAV paths generated by A- and D-optimality criteria, exposing some shortcomings of BFIM-based optimization.
- Three UAV path optimization algorithms tailored for joint self-localization and target tracking based on the A- and D-optimality criteria, and the closed-form projection algorithm in [20].

The paper is organized as follows. Section 2 describes the joint UAV self-localization and target tracking problem. The A- and D-optimality criteria are also introduced. In Section 3, the augmented state space model for Kalman filtering is formulated, leading to an extended Kalman filter estimator. Section 4 develops the UAV path optimization algorithms based on the A- and D-optimality criteria and an approximation of the recursive BFIM. The closed-form projection algorithm is also described. In Section 5, extensive simulation studies are presented to demonstrate the efficacy of the developed UAV path optimization algorithms in target localization and tracking. The concluding remarks are made in Section 6.

## 2. Problem Definition

**Figure 1:**
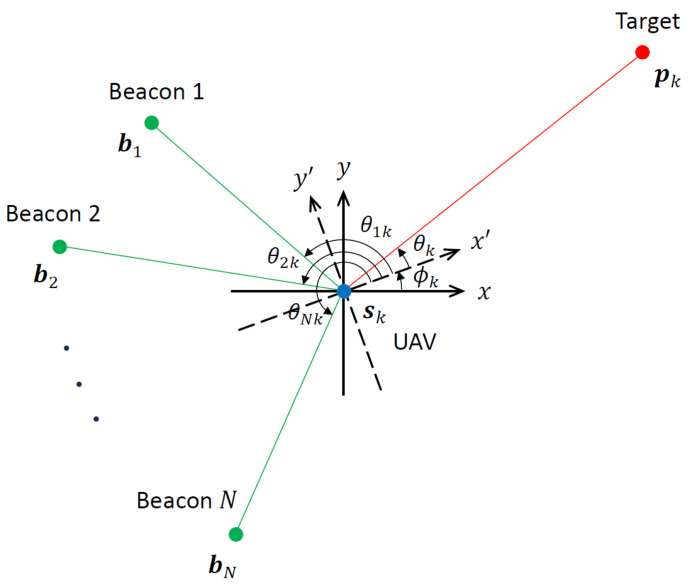
Geometry for UAV self-localization and target tracking using beacon bearings and the target AOA. The UAV location $s_{k}$, its orientation angle $\phi_{k}$ and target location $p_{k}$ are unknown and are to be estimated jointly from noisy beacon bearing and target AOA measurements.

The UAV self-localization and target tracking problem considered in this paper is depicted in Figure 1. The UAV uses the bearings from *N* beacons at a priori known locations $b_{i}={\left[b_{i1},b_{i2}\right]}^{T}$, i=1,…,N, in the global Cartesian coordinate system to estimate its own location $s_{k}={\left[s_{1k},s_{2k}\right]}^{T}$ at time k=0,1,2,… and the misalignment between its local coordinate system $x^{\prime }y^{\prime }$ and the global coordinate system xy, given by the orientation angle $\phi_{k}$. For an unambiguous solution, at least three beacons are required (N≥3). Furthermore, the beacons and the UAV must not be collinear or cocircular to avoid unobservable geometries with no unique solution [3]. The orientation angle represents platform vibrations, navigation errors, etc., and is usually time-varying. At time *k*, the beacon bearing angles are given by

(1) $$\theta_{ik}=\tan^{-1}\left(\frac{b_{i2}-s_{2k}}{b_{i1}-s_{1k}}\right)-\phi_{k},\;i=1,\ldots ,N$$

where $\tan^{-1}\left(\cdot \right)$ is the four-quadrant arctangent function.

The UAV collects angle-of-arrival (AOA) measurements from a manoeuvring target located at $p_{k}=\left[x_{k},y_{k}\right]$ in the global Cartesian coordinate system. The AOA in the absence of measurement noise is

(2) $$\theta_{k}=\tan^{-1}\left(\frac{y_{k}-s_{k2}}{x_{k}-s_{k1}}\right)-\phi_{k}.$$

Note that in both (1) and (2), the angles observed at the UAV are adjusted account for the orientation angle $\phi_{k}$.

A nearly constant velocity motion model [26] is assumed for the manoeuvring target. Accordingly, the dynamical equation for target state transitions is

(3) $$x_{t,k+1}=F_{t}x_{t,k}+n_{t,k},\;k=0,1,\ldots$$

where $x_{t,k}={\left[x_{k},{\dot{x}}_{k},y_{k},{\dot{y}}_{k}\right]}^{T}$ is the target state vector which contains the target location $p_{k}$ and its velocity $\left[{\dot{x}}_{k},{\dot{y}}_{k}\right]$ in global coordinates at time *k*. The state transition matrix is given by

(4) $$F_{t}=\left[\begin{array}{cc} A & 0\\ 0 & A \end{array}\right],\;A=\left[\begin{array}{cc} 1 & T\\ 0 & 1 \end{array}\right]$$

where *T* denotes the time interval in seconds between discrete-time instants *k*. In (3), $n_{t,k}$ is the process noise which accounts for unknown target manoeuvres and is a zero-mean white Gaussian random vector with covariance

(5) $$Q_{t}=\left[\begin{array}{cc} q_{tx}B & 0\\ 0 & q_{ty}B \end{array}\right],\;B=\left[\begin{array}{cc} T^{4}/4 & T^{3}/2\\ T^{3}/2 & T^{2} \end{array}\right]$$

where $q_{tx}$ and $q_{ty}$ are determined from the maximum target acceleration [26].

The main problem addressed in this paper is to determine an optimal UAV trajectory or flight path in the sense of optimizing a well-defined performance measure for target tracking. As the UAV needs to estimate its own location from beacon bearings while engaged in target tracking, the performance measure in question must invariably take into account the UAV self-localization performance. Poor self-localization will degrade target tracking by adding to the uncertainty in target AOA measurements. Thus, the coupling between self-localization and target tracking should be uncovered, which is achieved by an augmented Kalman filter.

To derive UAV path optimization algorithms, we adopt the A- and D-optimality criteria for experimental design [23,24,25]. The self-localization and target tracking problem is first cast into a Bayesian estimation framework using the extended Kalman filter (EKF). However, it should be stressed that the results derived in the paper are not restricted to the EKF and can be extended to other variants of the Kalman filter in a straightforward way. The optimal performance bound for the Kalman filter is given by the recursive Bayesian Fisher information matrix (BFIM) [22], which can be approximated to simplify its computation using readily available Kalman filter estimates.

The A-optimality criterion aims to minimize the trace of the inverse BFIM. As the inverse BFIM is the the Bayesian Cramer–Rao lower bound (BCRLB) (also known as the posterior Cramer–Rao lower bound), which is the theoretical lower bound on the covariance of any unbiased Bayesian estimator, the A-optimality criterion is equivalent to minimizing the bound on the mean-squared error (MSE). For a two-dimensional estimation problem, the A-optimality criterion minimizes the sum of squared major and minor axes of the error ellipse corresponding to the BCRLB.

The objective of the D-optimality criterion is to maximize the determinant of the BFIM, which is the same as minimizing the determinant of the BCRLB, given the inverse matrix relationship between the BFIM and BCRLB. Thus, the D-optimality criterion minimizes the product of squared major and minor axes of the error ellipse for the BCRLB, which is proportional to the area of the error ellipse in a two-dimensional estimation problem.

Both optimality criteria aim to reduce the size of the error ellipse for the BCRLB by minimizing either the sum or product of its squared semi-axes. They produce identical optimal geometries for some sensor modalities and specific scenarios, and completely different optimal geometries for others. For tracking problems where the optimal sensor path can only achieve incremental optimality, the two criteria have been observed to generate different optimal paths for a moving sensor (see, e.g., [20]).

## 3. Augmented State-Space Model for Self-Localization and Tracking

For joint UAV self-localization and target tracking using a Kalman filter, we consider an augmented state-space model, whereby the augmented state vector contains all the unknown parameters to be estimated

(6) $$x_{k}={\left[x_{k},{\dot{x}}_{k},y_{k},{\dot{y}}_{k},s_{1k},{\dot{s}}_{1k},s_{2k},{\dot{s}}_{2k},\phi_{k}\right]}^{T}.$$

The UAV state vector ${\left[s_{1k},{\dot{s}}_{1k},s_{2k},{\dot{s}}_{2k}\right]}^{T}$, which includes the UAV location $s_{k}$ and velocity ${\left[{\dot{s}}_{1k},{\dot{s}}_{2k}\right]}^{T}$, is part of the augmented state vector for self-localization purposes.

The dynamical equation for augmented state transitions is

(7) $$x_{k+1}=\underset{F}{\underset{︸}{\left[\begin{array}{ccc} F_{t} & 0 & 0\\ 0 & F_{s} & 0\\ 0 & 0 & \lambda \end{array}\right]}}x_{k}+n_{k}$$

where the state transition matrices for the target and UAV are identical ($F_{t}=F_{s}$), assuming a time-synchronized AOA and beacon bearing measurements, and the augmented process noise is

(8) $$n_{k}=\left[\begin{array}{c} n_{t,k}\\ n_{s,k}\\ n_{\phi ,k} \end{array}\right].$$

The individual process noise components for the target, UAV and orientation angle are $n_{t,k}∼N\left(0,Q_{t}\right)$ [see (3) and (5)], $n_{s,k}∼N\left(0,Q_{s}\right)$ and $n_{\phi ,k}∼N\left(0,\sigma_{\phi }^{2}\right)$, respectively, with

(9) $$Q_{s}=\left[\begin{array}{cc} q_{sx}B & 0\\ 0 & q_{sy}B \end{array}\right]$$

where $q_{sx}$ and $q_{sy}$ are the acceleration parameters for the UAV. Thus, the process noise for the augmented state space $n_{k}$ in (8) is a zero-mean white Gaussian random vector with covariance

(10) $$Q=\left[\begin{array}{ccc} Q_{t} & 0 & 0\\ 0 & Q_{s} & 0\\ 0 & 0 & \sigma_{\phi }^{2} \end{array}\right].$$

In (7), the orientation angle is modelled as a first-order autoregressive process

(11) $$\phi_{k+1}=\lambda \phi_{k}+n_{\phi ,k}$$

where 0<λ<1, so the variance of $\phi_{k}$ remains bounded at $\sigma_{\phi }^{2}/\left(1-\lambda^{2}\right)$.

The nonlinear measurement equation for the augmented state-space model is made up of target AOA and beacon bearing measurements collected by the UAV:

(12) $$z_{k}=\left[\begin{array}{c} {\hat{\theta }}_{k}\\ {\hat{\theta }}_{1k}\\ {\hat{\theta }}_{2k}\\ \vdots \\ {\hat{\theta }}_{Nk} \end{array}\right]=\underset{h(x_{k})}{\underset{︸}{\left[\begin{array}{c} h(x_{k})\\ h_{1}\left(x_{k}\right)\\ h_{2}\left(x_{k}\right)\\ \vdots \\ h_{N}\left(x_{k}\right) \end{array}\right]}}+w_{k}$$

where

(13) $$\begin{array}{c} h\left(x_{k}\right)=\tan^{-1}\left(\frac{y_{k}-s_{2k}}{x_{k}-s_{1k}}\right)-\phi_{k}\\ h_{i}\left(x_{k}\right)=\tan^{-1}\left(\frac{b_{i2}-s_{2k}}{b_{i1}-s_{1k}}\right)-\phi_{k},\;i=1,\ldots ,N \end{array}$$

and $w_{k}∼N\left(0,R\right)$ is the zero-mean white Gaussian measurement noise with a diagonal covariance matrix R of size (N+1)×(N+1). The diagonal entries of R contain the noise variances for the target AOA and beacon bearing measurements:

(14) $$R=\left[\begin{array}{cccc} \sigma_{\theta }^{2} & \; & & 0\\ \; & \sigma_{\theta_{1}}^{2} & \; & \\ \; & \; & \ddots & \\ 0 & \; & & \sigma_{\theta_{N}}^{2} \end{array}\right].$$

Equations (7) and (12) define the augmented state-space model for joint self-localization and target tracking. The measurement Equation (12) can be linearized by approximating the nonlinear functions in (13) by means of a truncated Taylor series expansion:

(15) $$\begin{array}{c} h\left(x_{k}\right)\approx h_{k}x_{k}\\ h_{i}\left(x_{k}\right)\approx h_{ik}x_{k} \end{array}$$

where

(16) $$\begin{array}{cc} h_{k} & =\frac{\partial h(x_{k})}{\partial x_{k}} \end{array}$$

(17) $$\begin{array}{c} =\left[-\frac{y_{k}-s_{2k}}{∥p_{k}-s_{k}{∥}^{2}},0,\frac{x_{k}-s_{1k}}{∥p_{k}-s_{k}{∥}^{2}},0,\frac{y_{k}-s_{2k}}{∥p_{k}-s_{k}{∥}^{2}},0,-\frac{x_{k}-s_{1k}}{∥p_{k}-s_{k}{∥}^{2}},0,-1\right] \end{array}$$

(18) $$\begin{array}{cc} \; & =\left[-\frac{\sin(\theta_{k}+\phi_{k})}{∥p_{k}-s_{k}∥},0,\frac{\cos(\theta_{k}+\phi_{k})}{∥p_{k}-s_{k}∥},0,\frac{\sin(\theta_{k}+\phi_{k})}{∥p_{k}-s_{k}∥},0,-\frac{\cos(\theta_{k}+\phi_{k})}{∥p_{k}-s_{k}∥},0,-1\right] \end{array}$$

and

(19) $$\begin{array}{cc} h_{ik} & =\frac{\partial h_{i}\left(x_{k}\right)}{\partial x_{k}} \end{array}$$

(20) $$\begin{array}{c} =\left[0,0,0,0,\frac{b_{i2}-s_{2k}}{∥b_{i}-s_{k}{∥}^{2}},0,-\frac{b_{i1}-s_{1k}}{∥b_{i}-s_{k}{∥}^{2}},0,-1\right] \end{array}$$

(21) $$\begin{array}{cc} \; & =\left[0,0,0,0,\frac{\sin(\theta_{ik}+\phi_{k})}{∥b_{i}-s_{k}∥},0,-\frac{\cos(\theta_{ik}+\phi_{k})}{∥b_{i}-s_{k}∥},0,-1\right]. \end{array}$$

Substituting (15) into (12) results in a linear approximation for the measurement equation:

(22) $$z_{k}=H_{k}x_{k}+w_{k},\;H_{k}=\left[\begin{array}{c} h_{k}\\ h_{1k}\\ h_{2k}\\ \vdots \\ h_{Nk} \end{array}\right]$$

which is used by the EKF to linearize the state space model [27].

The computational steps of the EKF for state estimation are summarized below:

State Prediction:
  (23) $$x_{k|k-1}=Fx_{k-1|k-1}\;\mathrm{mean}\;\mathrm{of}\;\mathrm{prior}$$
  (24) $$P_{k|k-1}=FP_{k-1|k-1}F^{T}+Q\;\mathrm{covariance}\;\mathrm{of}\;\mathrm{prior}$$
State Update:
  (25) $${\tilde{z}}_{k}=z_{k}-h\left(x_{k|k-1}\right)\;\mathrm{innovation}$$
  (26) $$G_{k}=P_{k|k-1}H_{k|k-1}^{T}{\left(H_{k|k-1}P_{k|k-1}H_{k|k-1}^{T}+R\right)}^{-1}\;\mathrm{Kalman}\;\mathrm{gain}$$
  (27) $$x_{k|k}=x_{k|k-1}+G_{k}{\tilde{z}}_{k}\;\mathrm{state}\;\mathrm{estimate}$$
  (28) $$P_{k|k}=\left(I-G_{k}H_{k|k-1}\right)P_{k|k-1}\;\mathrm{covariance}\;\mathrm{of}\;\mathrm{state}\;\mathrm{estimate}$$
  where $x_{k|k-1}$ is the state prediction at time *k* given all the measurements up to time k−1, and $x_{k|k}$ is the filtered state estimate at time *k* given all the measurements up to time *k*. $P_{k|k-1}$ and $P_{k|k}$ are the error covariance matrices for state prediction $x_{k|k-1}$ and filtered state estimate $x_{k|k}$, respectively, and $H_{k|k-1}$ is the Jacobian matrix $H_{k}$ in (22) evaluated at $x_{k}=x_{k|k-1}$:
  (29) $$H_{k|k-1}=\left[\begin{array}{c} h_{k|k-1}\\ h_{1,k|k-1}\\ h_{2,k|k-1}\\ \vdots \\ h_{N,k|k-1} \end{array}\right]={\left.H_{k}\right|}_{x_{k}=x_{k|k-1}}.$$
  The EKF recursively computes the Bayesian estimate for the state vector $x_{k|k}$ and its covariance $P_{k|k}$ using the Gaussian prior for the state available from the previous recursion, $x_{k|k-1}$ and $P_{k|k-1}$, and the measurements collected at the current recursion, $z_{k}$.

The EKF is initialized by the mean and covariance of the state at k=0:

(30) $$\begin{array}{c} x_{0|-1}=E\left\{x_{0}\right\} \end{array}$$

(31) $$\begin{array}{c} P_{0|-1}=\mathrm{Cov}\left\{x_{0}\right\}. \end{array}$$

Furthermore, the EKF requires prior knowledge of the process and measurement noise covariances, Q and R, respectively.

## 4. UAV Path Optimization

### 4.1. Estimation Bound for the EKF

To develop UAV path optimization algorithms based on the A- and D-optimality criteria, we require a performance bound on the covariance of the filtered state estimates computed by the EKF. The BCRLB provides this bound. For the EKF, the BFIM, which is the inverse of the BCRLB, is recursively computed using [22]

(32) $$\Phi_{k}={\left(F\Phi_{k-1}F^{T}+Q\right)}^{-1}+E_{x_{k}}\left\{H_{k}^{T}R^{-1}H_{k}\right\}$$

where $H_{k}$ is the Jacobian matrix in (22) calculated at the true target state $x_{k}$. Equation (32) is the sum of prior information ${\left(F\Phi_{k-1}F^{T}+Q\right)}^{-1}$ available from the previous recursion and contributions from measurements at the current recursion $E_{x_{k}}\left\{H_{k}^{T}R^{-1}H_{k}\right\}$. Under mild conditions, (32) can be approximated as

(33) $$\begin{array}{cc} \Phi_{k} & \approx P_{k|k}^{-1}\\ \; & \approx P_{k|k-1}^{-1}+H_{k|k-1}^{T}R^{-1}H_{k|k-1}\\ & \approx P_{k|k-1}^{-1}+\underset{I_{k}}{\underset{︸}{\frac{1}{\sigma_{\theta }^{2}}h_{k|k-1}^{T}h_{k|k-1}}}+\underset{J_{k}}{\underset{︸}{\sum_{i=1}^{N}\frac{1}{\sigma_{\theta_{i}}^{2}}h_{i,k|k-1}^{T}h_{i,k|k-1}}} \end{array}$$

where only the readily available EKF estimates are used, thereby avoiding computationally expensive Monte Carlo simulations to compute the expectation over $x_{k}$ on the right-hand side of (32). Referring to (21), we note that in (33), the contribution of beacon bearing measurements is a sparse block matrix

(34) $$\begin{array}{l} \begin{array}{cccccc} & & 4 & & 5 & \end{array}\\ J_{k}=\left[\begin{array}{cc} 0 & 0\\ 0 & * \end{array}\right]\begin{array}{c} 4\\ 5 \end{array} \end{array}$$

where only the 5×5 lower diagonal submatrix has nonzero entries.

Let $\Sigma_{k}=\Phi_{k}^{-1}$ denote the recursive BCRLB which gives the lower bound on the covariance of $x_{k|k}$ for any unbiased estimate. Writing $\Phi_{k}$ and $\Sigma_{k}$ in a block matrix form:

(35) $$\begin{array}{l} \begin{array}{cccccc} & & 4 & & 5 & \end{array}\\ \Phi_{k}=\left[\begin{array}{cc} A_{k} & B_{k}\\ C_{k} & D_{k} \end{array}\right]\begin{array}{c} 4\\ 5 \end{array} \end{array}\;\begin{array}{l} \begin{array}{ccccccccc} & & & & & 4 & & 5 & \end{array}\\ \Sigma_{k}=\Phi_{k}^{-1}=\left[\begin{array}{cc} \Sigma_{11,k} & \Sigma_{12,k}\\ \Sigma_{21,k} & \Sigma_{22,k} \end{array}\right]\begin{array}{c} 4\\ 5 \end{array} \end{array}$$

we have

(36) $$\Sigma_{11,k}=A_{k}^{-1}+A_{k}^{-1}B_{k}{\left(D_{k}-C_{k}A_{k}^{-1}B_{k}\right)}^{-1}C_{k}A_{k}^{-1}$$

which is the recursive BCRLB for target tracking. The second term on the right-hand side of (36) represents the contribution of self-localization to the target tracking performance, arising from the coupling between self-localization and target tracking, as evidenced by nonzero submatrices $B_{k}$ and $C_{k}$ in $\Phi_{k}$. As our ultimate objective is to optimize the target tracking performance by means of appropriate UAV manoeuvres, the 4×4 submatrix of the BCRLB, $\Sigma_{11,k}$, will be the focus of attention. This is different from previous work, where the entire $\Sigma_{k}$ was used [16,21].

In (33), the contributions of the target AOA and beacon bearing measurements ($I_{k}$ and $J_{k}$, respectively) to the recursive BFIM are influenced by several factors, the most obvious of which are the angle noise variances $\sigma_{\theta }^{2}$ and $\sigma_{\theta_{i}}^{2}$, and the distances of the UAV from the target and beacons (i.e., $∥p_{k}-s_{k}∥$ and $∥b_{i}-s_{k}∥$). For a moment, consider the contribution of $I_{k}$ individually in terms of the target AOA noise variance and distance of the UAV from the target. Referring to (18) and (33), it is clear that the product $\sigma_{\theta }^{2}∥p_{k}-s_{k}∥$ determines the extent to which the target range $∥p_{k}-s_{k}∥$ contributes to the recursive BFIM. The smaller $\sigma_{\theta }^{2}∥p_{k}-s_{k}∥$, the larger the contribution of $I_{k}$ to the recursive BFIM will be. Specifically, if $\sigma_{\theta }^{2}$ is very small, decreasing $∥p_{k}-s_{k}∥$ will not impact the recursive BFIM significantly. This means that the distance reduction between the UAV and the target will not be prioritized, instead favouring an almost circular UAV trajectory around the target. Conversely, for a large $\sigma_{\theta }^{2}$, reducing the distance between the UAV and the target will have a significant impact on the recursive BFIM, thereby guiding the UAV closer to the target. This observation exposes a potential pitfall for A- and D-optimality-based UAV path optimization algorithms built on the recursive BFIM when the angle measurements are very accurate, for example, thanks to a high-precision imaging camera onboard the UAV.

The allowable maximum target distance from the beacons is an important design parameter, which determines the size of the coverage area to achieve a minimum target tracking performance that is acceptable. As the UAV becomes close to the target, the target tracking performance improves if $I_{k}$ is considered alone. However, if the close proximity between the UAV and target translates into a significant increase in the UAV distance from the beacons, the UAV self-localization performance will degrade as the contribution of $J_{k}$ to $\Phi_{k}$, which is captured in the submatrix of $\Phi_{k}$, $D_{k}$, diminishes with an increased $∥b_{i}-s_{k}∥$ [see (21) and (33)]. In (35), the submatrices $A_{k}$, $B_{k}$ and $C_{k}=B_{k}^{T}$ are not affected by changes in $∥b_{i}-s_{k}∥$, and only $D_{k}$ depends directly on $∥b_{i}-s_{k}∥$. Rewriting (36) as

(37) $$\Sigma_{11,k}={\left(A_{k}-B_{k}D_{k}^{-1}B_{k}^{T}\right)}^{-1}$$

it can be shown that $A_{k}≻B_{k}D_{k}^{-1}B_{k}^{T}$ (i.e., $A_{k}-B_{k}D_{k}^{-1}B_{k}^{T}$ is positive definite) for a positive-definite $\Phi_{k}$, and a decrease in the eigenvalues of $D_{k}$ resulting from a significant increase in $∥b_{i}-s_{k}∥$ will lead to an increase in both the trace and determinant of the covariance matrix $\Sigma_{11,k}$. This in turn implies a poor tracking performance. In such a situation, the UAV path optimization will attempt to maximize the sum of contributions from $I_{k}$ and $J_{k}$ by balancing the UAV distances from the target and beacons rather than guiding the UAV close to the target, which will likely result in a UAV trajectory somewhere in between the target and the beacon cluster. In general, the farther away the target is from the beacons, the worse the tracking performance will be. The acceptable minimum tracking performance, such as the minimum mean-square error for target location estimates, dictates how far the target can be from the beacons.

### 4.2. UAV Path Optimization Algorithm Based on the A-Optimality Criterion

The objective of the A-optimality criterion is to minimize the trace of the recursive BCRLB for target location estimates over UAV waypoints:

(38) $$s_{k|k-1}^{*}=\underset{s_{k|k-1}\in S_{k}}{arg min}\;J_{A,k}\left(s_{k|k-1}\right)$$

where $s_{k|k-1}^{*}$ is the next optimal waypoint for the UAV, $J_{A,k}\left(s_{k|k-1}\right)$ is the trace of the BCRLB for target location estimates, and $S_{k}$ is the set of permissible waypoints for the UAV at time *k*, compliant with the maximum turnrate, UAV speed and the time interval for angle measurements *T*. Defining the BCRLB for target location estimates as

(39) $$\Sigma_{p,k}=\left[\begin{array}{cc} \sigma_{11,k} & \sigma_{13,k}\\ \sigma_{31,k} & \sigma_{33,k} \end{array}\right]$$

which is related to $\Sigma_{11,k}$ via

(40) $$\Sigma_{11,k}={\left[\begin{array}{cccc} \sigma_{11,k} & * & \sigma_{13,k} & *\\ & * & * & *\\ \sigma_{31,k} & * & \sigma_{33,k} & *\\ & * & * & * \end{array}\right]}_{4\times 4}$$

we have

(41) $$J_{A,k}\left(s_{k|k-1}\right)=\mathrm{tr}\Sigma_{p,k}=\sigma_{11,k}+\sigma_{33,k}.$$

**Figure 2:**
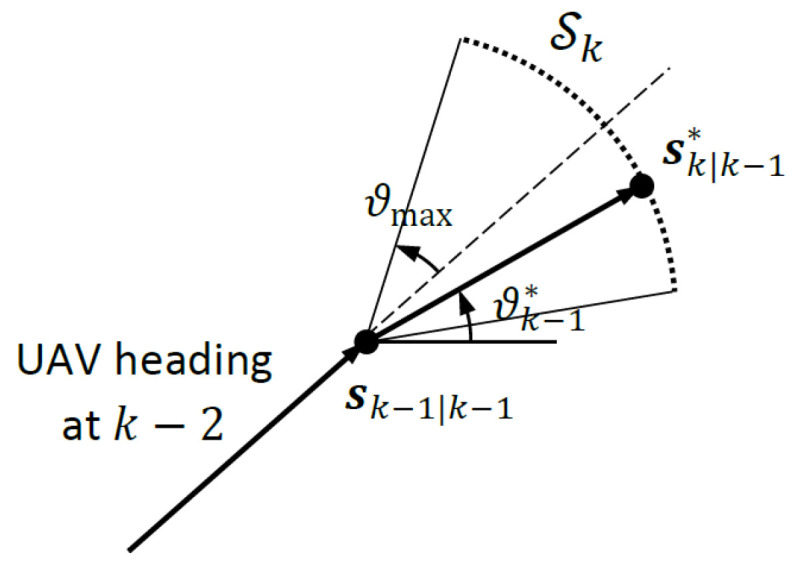
Illustration of the set of permissible waypoints $S_{k}$ and maximum turnrate $\vartheta_{\max}$.

As illustrated in Figure 2, $S_{k}$ contains equally spaced discrete points on an arc that is centred at the current heading of the UAV subtending an angle of $2\vartheta_{\max}$, where $\vartheta_{\max}$ is the maximum turnrate for the UAV. The members of $S_{k}=\left\{s_{1k},s_{2k},\ldots ,s_{Mk}\right\}$ must satisfy

(42) $$s_{ik}-s_{k-1|k-1}=s\nu \left(\vartheta_{k-1}\right)\;\mathrm{and}\;\left|\Delta \vartheta_{k-1}\right|<\vartheta_{\max},\;i=1,2,\ldots ,M$$

where *s* is the distance between successive waypoints (the radius of the maximum turnrate arc that contains the members of $S_{k}$), $\nu (\vartheta_{k-1})$ is the unit vector for the heading at time k−1:

(43) $$\nu \left(\vartheta_{k-1}\right)=\left[\begin{array}{c} \cos\vartheta_{k-1}\\ \sin\vartheta_{k-1} \end{array}\right]$$

and $\Delta \vartheta_{k-1}=\vartheta_{k-1}-\vartheta_{k-2}$ is the change in heading angle at time k−1.

The optimal heading towards the next waypoint is calculated relative to the estimated UAV location $s_{k-1|k-1}$ and orientation angle $\phi_{k-1|k-1}$ computed by the EKF. For simulation purposes, it is necessary to determine the actual UAV motion in global coordinates derived from path optimization and EKF estimates. This is achieved by mapping the computed waypoints to the global Cartesian coordinate system using

(44) $$\begin{array}{c} s_{k-1|k-1}⟶s_{k-1}\\ s_{k|k-1}^{*}=s_{k-1|k-1}+s\nu \left(\vartheta_{k-1}^{*}\right)⟶s_{k}=s_{k-1}+s\nu \left(\vartheta_{k-1}^{*}+\underset{\begin{array}{c} \mathrm{orientation}\;\mathrm{estimation}\\ \mathrm{error} \end{array}}{\underset{︸}{\phi_{k-1}-\phi_{k-1|k-1}}}\right) \end{array}$$

where $\vartheta_{k-1}^{*}$ is the optimal UAV heading at time k−1 obtained from (38).

The computational steps of the A-optimality criterion-based path optimization algorithm are summarized below:

1. Given *s* and $\vartheta_{\max}$, determine the equally spaced *M* members of $S_{k}$ according to (42) and Figure 2.
2. Re-calculate $H_{k|k-1}$ and $\Phi_{k}$ in (33) for each candidate waypoint $s_{k|k-1}\in S_{k}$.
3. Re-calculate $\Sigma_{11,k}$ for each $\Phi_{k}$ in Step 2 using (35) and (36).
4. Calculate $J_{A,k}\left(s_{k|k-1}\right)$ for each $\Sigma_{11,k}$ in Step 3, and find $s_{k|k-1}$ for which $J_{A,k}\left(s_{k|k-1}\right)$ is minimized.

### 4.3. UAV Path Optimization Algorithm Based on the D-Optimality Criterion

The D-optimality criterion maximizes the determinant of the recursive BFIM for target location estimates over UAV waypoints. In terms of the recursive BCRLB, this is equivalent to

(45) $$s_{k|k-1}^{*}=\min_{s_{k|k-1}\in S_{k}}\;J_{D,k}\left(s_{k|k-1}\right)$$

where $J_{D,k}\left(s_{k}\right)$ is the determinant of $\Sigma_{p,k}$ in (39):

(46) $$J_{D,k}\left(s_{k}\right)=\det\left(\Sigma_{p,k}\right)=\sigma_{11,k}\sigma_{33,k}-\sigma_{31,k}\sigma_{13,k}.$$

The set of permissible waypoints $S_{k}$ is defined in (42). The optimal waypoint $s_{k|k-1}^{*}$ found in (45) is mapped to the global coordinates according to (44).

The computational steps of the D-optimality criterion-based path optimization algorithm are

1. Given *s* and $\vartheta_{\max}$, determine the equally spaced *M* members of $S_{k}$ according to (42) and Figure 2.
2. Re-calculate $H_{k|k-1}$ and $\Phi_{k}$ in (33) for each candidate waypoint $s_{k|k-1}\in S_{k}$.
3. Re-calculate $\Sigma_{11,k}$ for each $\Phi_{k}$ in Step 2 using (35) and (36).
4. Calculate $J_{D,k}\left(s_{k|k-1}\right)$ for each $\Sigma_{11,k}$ in Step 3, and find $s_{k|k-1}$ for which $J_{D,k}\left(s_{k|k-1}\right)$ is minimized.

### 4.4. Modified Projection Algorithm

A major criticism of the A- and D-optimality-based UAV path optimization algorithms presented in the previous two subsections is that they require a numerical search over *M* candidate waypoints in the set $S_{k}$ at every time instant *k*. This can prove computationally expensive or even prohibitive in practice. It is possible to develop an alternative closed-form approach, dispensing with an exhaustive numerical search by exploiting the knowledge of what would constitute an optimal geometry at a given time *k* if the UAV could be moved anywhere with no constraints on the distance between successive waypoints *s*. In our previous work, we showed that for AOA localization, the optimal sensor location is along the line extension of the minor axis of the Gaussian prior covariance error ellipse in the 2D plane [20]. Using the EKF estimates, this means that an optimal next waypoint for the UAV is ideally on a line overlapping the minor axis of the predicted state estimate covariance corresponding to the target location. However, this is not guaranteed to be achievable as the UAV motion is restricted by speed and turnrate constraints. Therefore, the proposed approach is to move the UAV to a waypoint where it is closer to the line extension of the minor axis. This idea was exploited in [20] to devise an optimization method called the projection algorithm. In this subsection, we show how to modify it so that it can be applied to the UAV path optimization problem for self-localization and target tracking.

To begin with, define the covariance matrix for the predicted target location estimate (covariance of the prior):

(47) $$P_{\mathrm{loc},k|k-1}=\left[\begin{array}{cc} p_{k|k-1}\left(1,1\right) & p_{k|k-1}\left(1,3\right)\\ p_{k|k-1}\left(3,1\right) & p_{k|k-1}\left(3,3\right) \end{array}\right]$$

where $P_{k|k-1}=\left\{p_{k|k-1}\left(i,j\right)\right\}$ is the covariance of state prediction $x_{k|k-1}$ which includes all the state variables. The state prediction for the target location gives the mean of the prior:

(48) $$x_{\mathrm{loc},k|k-1}=\left[\begin{array}{c} x_{k|k-1}\\ y_{k|k-1} \end{array}\right]$$

where

(49) $$x_{k|k-1}={\left[x_{k|k-1},{\dot{x}}_{k|k-1},y_{k|k-1},{\dot{y}}_{k|k-1},\ldots \right]}^{T}.$$

**Figure 3:**
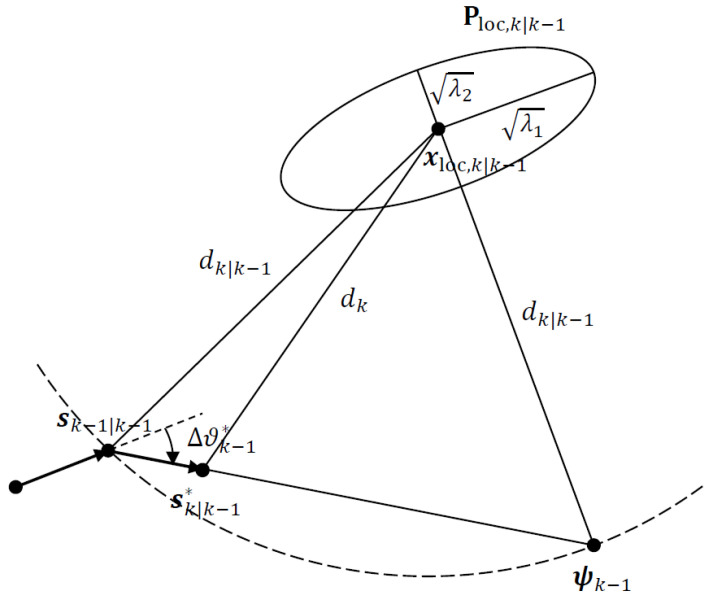
Modified projection algorithm for UAV path optimization.

Given the Gaussian prior $N(x_{\mathrm{loc},k|k-1},P_{\mathrm{loc},k|k-1})$ and the UAV location estimate $s_{k-1|k-1}$, available from the previous EKF recursion at time k−1, the projection algorithm guides the UAV towards the line extension of the minor axis of the Gaussian prior at time *k*, subject to the maximum turnrate constraint. Figure 3 illustrates the operation of the projection algorithm. As the prior for the target location estimate is extracted from EKF state prediction, the coupling between self-localization and target tracking still affects the optimal UAV path decisions as the prior is updated recursively.

The projection algorithm proceeds as follows:

1. Given $s_{k-1|k-1}$, $x_{\mathrm{loc},k|k-1}$, $P_{\mathrm{loc},k|k-1}$, $\vartheta_{\max}$, $\vartheta_{k-2}$ and *s*, calculate the minor axis of the error ellipse of $P_{\mathrm{loc},k|k-1}$ from the eigenvector of $P_{\mathrm{loc},k|k-1}$ associated with its smallest eigenvalue.
2. Calculate the predicted target range $d_{k|k-1}=∥x_{\mathrm{loc},k|k-1}-s_{k-1|k-1}∥$.
3. Find $\psi_{k-1}$, the intersection of the line extension of the minor axis with the circle of radius $d_{k|k-1}$ centred at $x_{\mathrm{loc},k|k-1}$, closest to $s_{k-1|k-1}$.
4. Calculate the waypoint vector $\Delta s_{k-1}=s\left(\psi_{k-1}-s_{k-1|k-1}\right)/∥\psi_{k-1}-s_{k-1|k-1}∥$ and the optimal heading angle $\vartheta_{k-1}^{*}=∠\Delta s_{k-1}$
5. Find the optimal heading change $\Delta \vartheta_{k-1}^{*}=\vartheta_{k-1}^{*}-\vartheta_{k-2}$.
6. If $|\Delta \vartheta_{k-1}^{*}|<\vartheta_{\max}$, then the next optimal waypoint is $s_{k|k-1}^{*}=s_{k-1|k-1}+\Delta s_{k-1}$. Otherwise, restrict the heading angle for $\Delta s_{k-1}$ to the maximum turnrate: $\vartheta_{k-1}^{*}=\vartheta_{k-2}+\mathrm{sign}\left(\Delta \vartheta_{k-1}^{*}\right)\vartheta_{\max}$.
7. Find the actual UAV location $s_{k}$ using (44).

**Figure 4:**
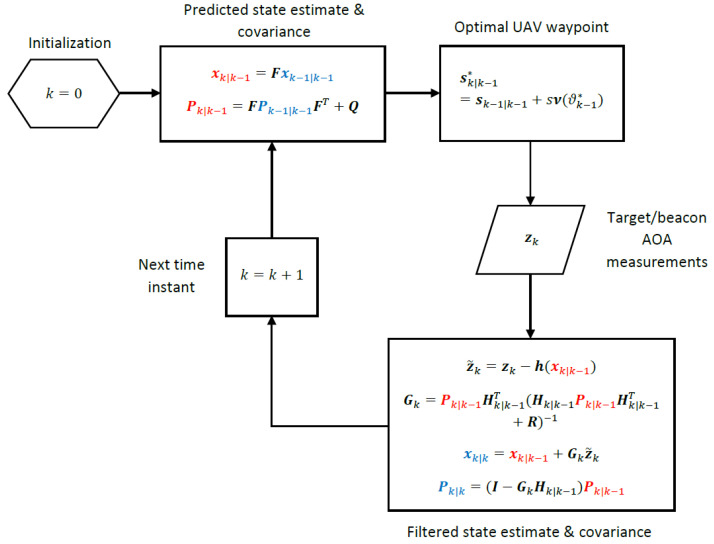
Computational architecture for UAV path optimization and target tracking. The A-optimality, D-optimality and projection algorithms differ in the way $\vartheta_{k-1}^{*}$, k=1,2,…, is computed.

The UAV path optimization algorithms derived in this section all have the same computational architecture, which is depicted in Figure 4. The algorithms only differ in the way the optimal heading angle $\vartheta_{k-1}^{*}$, k=1,2,…, is computed. The A- and D-optimality algorithms use a numerical search over permissible sets of candidate waypoints given by (38) and (45), respectively, and the projection algorithm incorporates a closed-form geometric solution (see Steps 4–7 above) to find the optimal heading.

## 5. Simulation Studies

This section presents numerical simulation examples to demonstrate the performance of the UAV path optimization algorithms for self-localization and target tracking developed in Section 4. In the simulations, two scenarios for a stationary (non-moving) target and a manoeuvring target are considered. A comparison with alternative approaches is also presented towards the end of the section.

For the stationary target, the unknown target location is a Gaussian random vector

(50) $$p_{0}∼N\left(\left[\begin{array}{c} -10\\ -5 \end{array}\right],\left[\begin{array}{cc} 9.2500 & -9.0933\\ -9.0933 & 19.7500 \end{array}\right]\right)\;\mathrm{km}.$$

The process noise is $n_{t,k}=0$ with $q_{tx}=q_{ty}=0$ in (3) and (5), and the initial target velocity is ${\left[{\dot{x}}_{0},{\dot{y}}_{0}\right]}^{T}=0$ in (3), as the target is not moving.

The manoeuvring target’s initial location is also given by (50). The target acceleration parameters are $q_{tx}=q_{ty}=10^{-10}$ km^2^/s^4^. The initial target velocity is ${\left[{\dot{x}}_{0},{\dot{y}}_{0}\right]}^{T}={\left[2.5\times 10^{-3},2.5\times 10^{-3}\right]}^{T}$ km/s, which corresponds to 12.7279 km/h.

The Gaussian prior for the initial UAV location is

(51) $$s_{0}∼N\left(\left[\begin{array}{c} -36.8116\\ -27.4976 \end{array}\right],\left[\begin{array}{cc} 10.3015 & -1.7101\\ -1.7101 & 19.6985 \end{array}\right]\right)\;\mathrm{km}.$$

The distance between the mean initial target and UAV locations is $∥E\left\{p_{0}\right\}-E\left\{s_{0}\right\}∥=35$ km. The UAV moves with a constant speed of 0.025 km/s (90 km/h) and a maximum turnrate of $\vartheta_{\max}=3^{\circ }$/s. Its initial heading in the global coordinate system is $\vartheta_{0}=0^{\circ }$. The orientation angle for the UAV, $\phi_{k}$, in (11) has the parameters λ=0.8, $\phi_{0}=10^{\circ }$ and $\sigma_{\phi }=2^{\circ }$. To facilitate UAV self-localization, four beacons are used (N=4) located at $b_{1}={\left[-45,-45\right]}^{T}$ km, $b_{2}={\left[45,-45\right]}^{T}$ km, $b_{3}={\left[-45,45\right]}^{T}$ km and $b_{4}={\left[45,45\right]}^{T}$ km.

The EKF is initialized by

(52) $$x_{0|-1}=\left[\begin{array}{c} x_{0|-1}\\ {\dot{x}}_{0|-1}\\ y_{0|-1}\\ {\dot{y}}_{0|-1}\\ s_{1,0|-1}\\ {\dot{s}}_{1,0|-1}\\ s_{2,0|-1}\\ {\dot{s}}_{2,0|-1}\\ \phi_{0|-1} \end{array}\right]=\left[\begin{array}{c} -10\\ 0\\ -5\\ 0\\ -36.8116\\ 0\\ -27.4976\\ 0\\ 10^{\circ } \end{array}\right],\;P_{0|-1}=\left[\begin{array}{ccc} P_{t,0|-1} & \; & 0\\ \; & P_{s,0|-1} & \\ 0 & \; & \sigma_{\phi }^{2} \end{array}\right]$$

where ${\left[x_{0|-1},y_{0|-1}\right]}^{T}=E\left\{p_{0}\right\}$, ${\left[s_{1,0|-1},s_{2,0|-1}\right]}^{T}=E\left\{s_{0}\right\}$, $\phi_{0|-1}=\phi_{0}$, and the diagonal block submatrices of $P_{0|-1}$ are

(53) $$P_{t,0|-1}=\left[\begin{array}{cccc} 9.2500 & 0 & -9.0933 & 0\\ 0 & 0 & 0 & 0\\ -9.0933 & 0 & 19.7500 & 0\\ 0 & 0 & 0 & 0 \end{array}\right],\;P_{s,0|-1}=\left[\begin{array}{cccc} 10.3015 & 0 & -1.7101 & 0\\ 0 & 0 & 0 & 0\\ -1.7101 & 0 & 19.6985 & 0\\ 0 & 0 & 0 & 0 \end{array}\right]$$

which are obtained from the Gaussian priors for the initial target and UAV locations in (50) and (51).

For UAV self-localization, the UAV acceleration parameters in (9) are set equal to $q_{sx}=q_{sy}=10^{-6}$ km^2^/s^4^. The time interval between angle measurements is T=10 s. The standard deviations for target AOA and beacon bearing measurement noise are assumed to be identical with $\sigma =\sigma_{\theta }=\sigma_{\theta_{1}}=\cdots =\sigma_{\theta_{4}}$, and are chosen from the set $\left\{0.1^{\circ },1^{\circ },2^{\circ }\right\}$, covering high- to low-precision angle measurements. The diagonal measurement covariance matrix R is constructed from the target AOA and beacon bearing noise variances according to (14).

**Figure 5:**
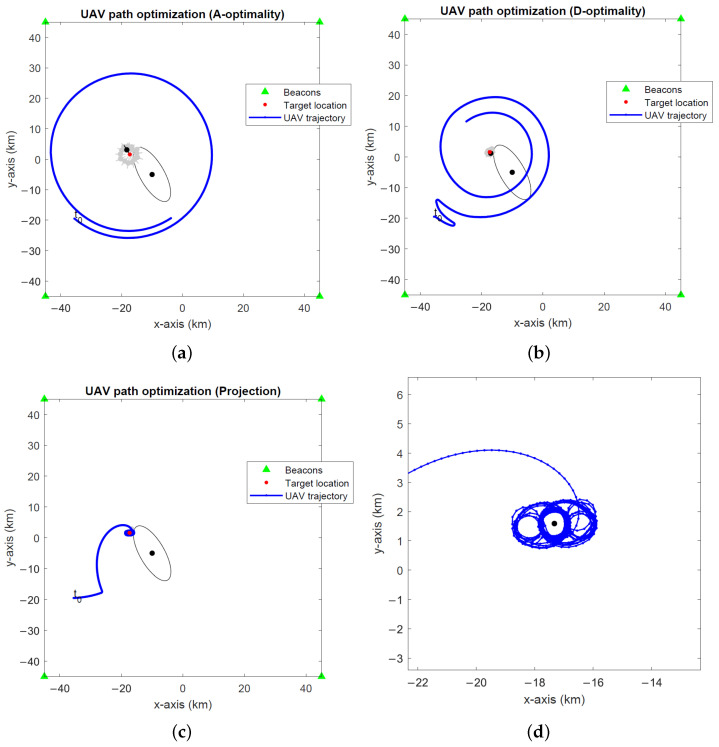
Optimal UAV paths for a stationary target at $\sigma =0.1^{\circ }$ computed by the (**a**) A-optimality algorithm, (**b**) D-optimality algorithm and (**c**) projection algorithm. (**d**) A close-up of the projection algorithm. The initial UAV location is marked with “$t_{0}$”. Black dots and lines indicate the 2-σ error ellipses for initial and final EKF target location estimates. Gray dots and lines show the 2-σ error ellipses for intermediate EKF estimates.

**Figure 6:**
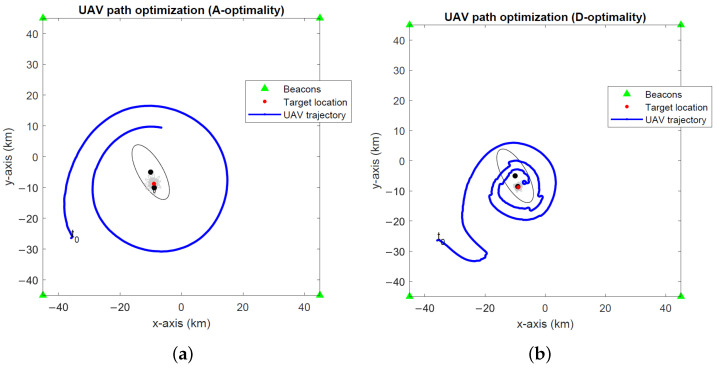

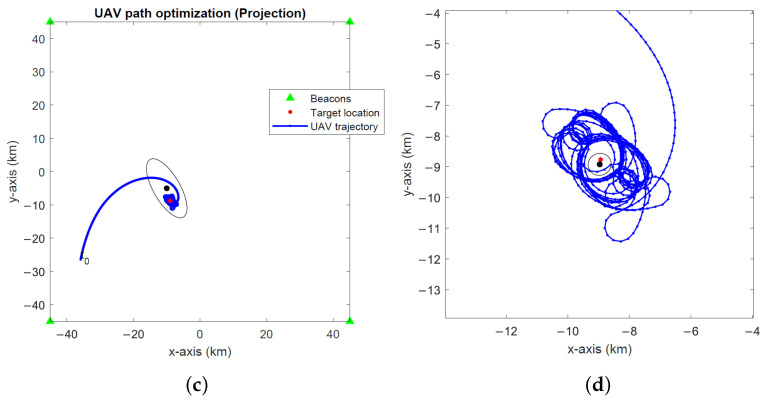
Optimal UAV paths for a stationary target at $\sigma =1^{\circ }$ computed by the (**a**) A-optimality algorithm, (**b**) D-optimality algorithm and (**c**) projection algorithm. (**d**) A close-up of the projection algorithm. The initial UAV location is marked with “$t_{0}$”. Black dots and lines indicate the 2-σ error ellipses for initial and final EKF target location estimates. Gray dots and lines show the 2-σ error ellipses for intermediate EKF estimates.

**Figure 7:**
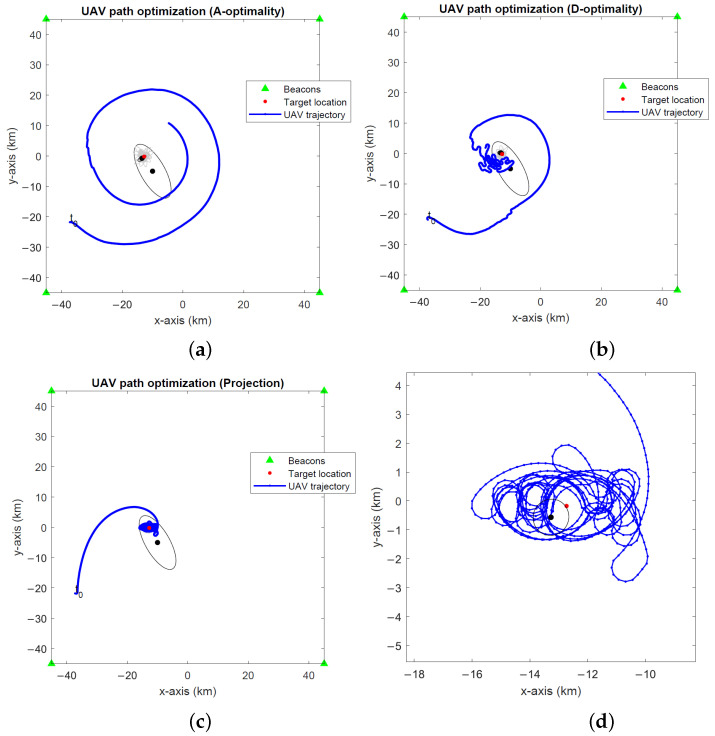
Optimal UAV paths for a stationary target at $\sigma =2^{\circ }$ computed by the (**a**) A-optimality algorithm, (**b**) D-optimality algorithm and (**c**) projection algorithm. (**d**) A close-up of the projection algorithm. The initial UAV location is marked with “$t_{0}$”. Black dots and lines indicate the 2-σ error ellipses for initial and final EKF target location estimates. Gray dots and lines show the 2-σ error ellipses for intermediate EKF estimates.

Figure 5, Figure 6 and Figure 7 show the optimal UAV paths for a stationary target in the global coordinates computed by the UAV path optimization algorithms based on the A- and D-optimality criteria and the projection algorithm at three different noise levels $\sigma \in \{0.1^{\circ },1^{\circ },2^{\circ }\}$. The A- and D-optimality-based UAV path optimization algorithms use a waypoint search set $S_{k}$ with M=10 members. The simulations run for 800 EKF recursions for a single realization of the random processes for target location, orientation angle and angle measurement noise. The initial UAV location is indicated with “$t_{0}$”. The 2-σ error ellipses for the target location on initialization and the final EKF estimate are shown by black ellipses. The 2-σ error ellipses for intermediate EKF estimates of the target location computed at every 50 recursions are shown by the gray ellipses. We observe that the A-optimality algorithm tends to circle around the target in favour of approaching it, which becomes even more pronounced at small noise (see Figure 5a). This observation is in agreement with an earlier remark made in Section 4.1 in relation to the relative insensitivity of the recursive BFIM to target distance reduction at small noise levels. The D-optimality algorithm circles around the target before approaching the target. The projection algorithm approaches the target much faster than the other two algorithms, clearly prioritizing target range reduction. This makes it the algorithm of choice, particularly in low-noise scenarios.

**Figure 8:**
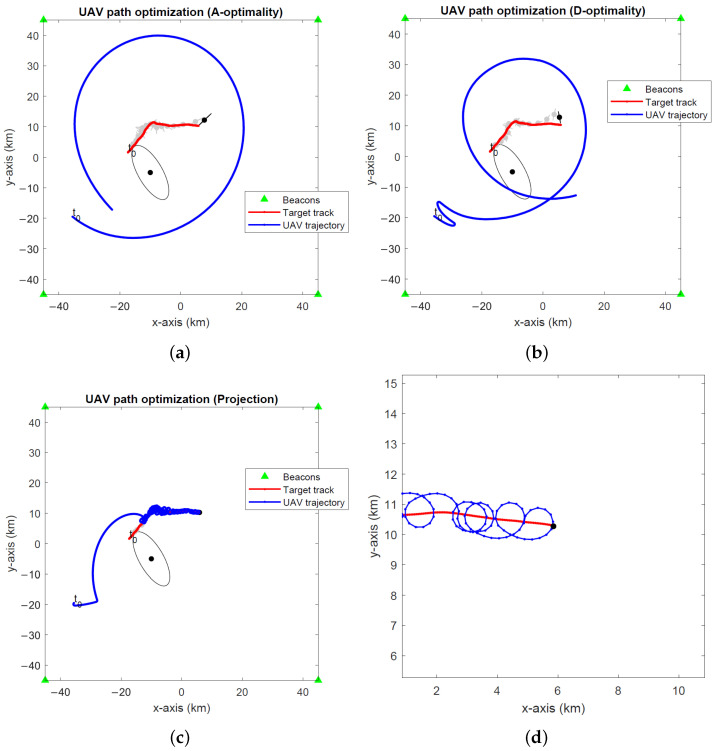
Optimal UAV paths for target tracking ($\sigma =0.1^{\circ }$) computed by the (**a**) A-optimality algorithm, (**b**) D-optimality algorithm and (**c**) projection algorithm. (**d**) A close-up of the projection algorithm. The initial UAV and target locations are marked with “$t_{0}$”. Black dots and lines indicate the 2-σ error ellipses for initial and final EKF target location estimates. Gray dots and lines show the 2-σ error ellipses for intermediate EKF estimates.

**Figure 9:**
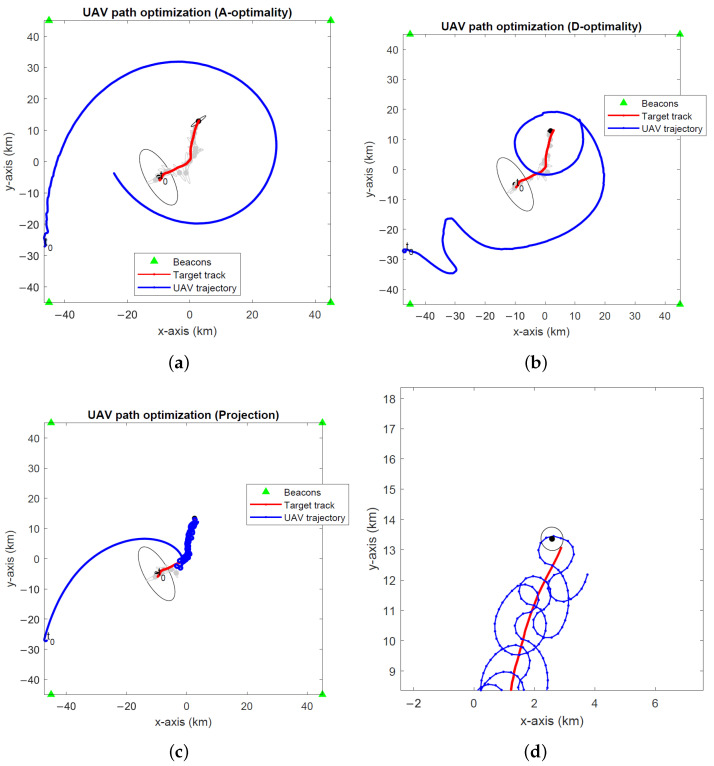
Optimal UAV paths for target tracking ($\sigma =1^{\circ }$) computed by the (**a**) A-optimality algorithm, (**b**) D-optimality algorithm and (**c**) projection algorithm. (**d**) A close-up of the projection algorithm. The initial UAV and target locations are marked with “$t_{0}$”. Black dots and lines indicate the 2-σ error ellipses for initial and final EKF target location estimates. Gray dots and lines show the 2-σ error ellipses for intermediate EKF estimates.

**Figure 10:**
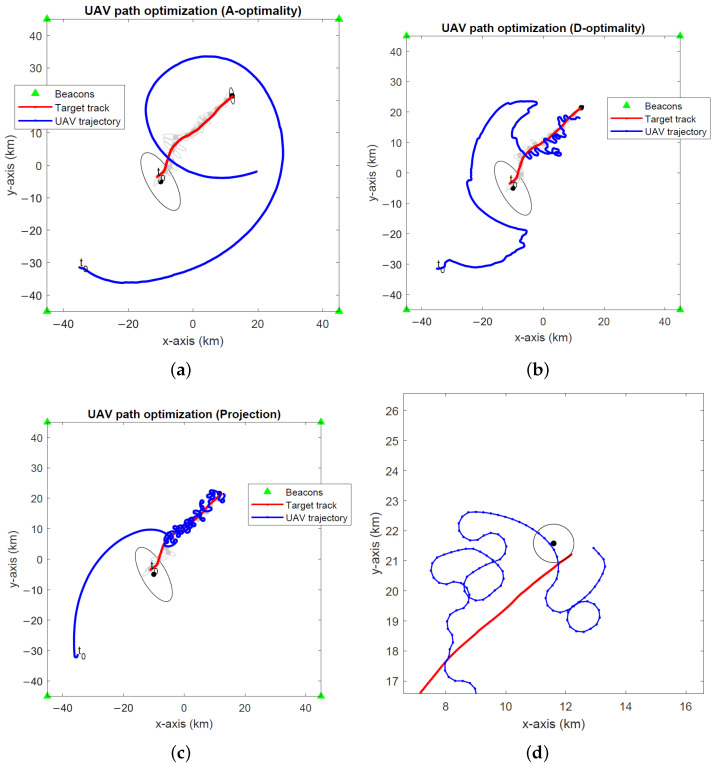
Optimal UAV paths for target tracking ($\sigma =2^{\circ }$) computed by the (**a**) A-optimality algorithm, (**b**) D-optimality algorithm and (**c**) projection algorithm. (**d**) A close-up of the projection algorithm. The initial UAV and target locations are marked with “$t_{0}$”. Black dots and lines indicate the 2-σ error ellipses for initial and final EKF target location estimates. Gray dots and lines show the 2-σ error ellipses for intermediate EKF estimates.

The optimal UAV paths for target tracking in the global coordinates computed by the A- and D-optimality UAV path optimization algorithms and the projection algorithm are depicted in Figure 8, Figure 9 and Figure 10 for noise levels $\sigma \in \{0.1^{\circ },1^{\circ },2^{\circ }\}$. The initial UAV and target locations are indicated with “$t_{0}$”. We make similar observations to the stationary target case in the previous simulations that the A-optimality algorithm tends to circle around the target more than the other two algorithms. The projection algorithm exhibits a direct approach towards the target followed by close manoeuvres around the target dictated by the maximum turnrate.

**Figure 11:**
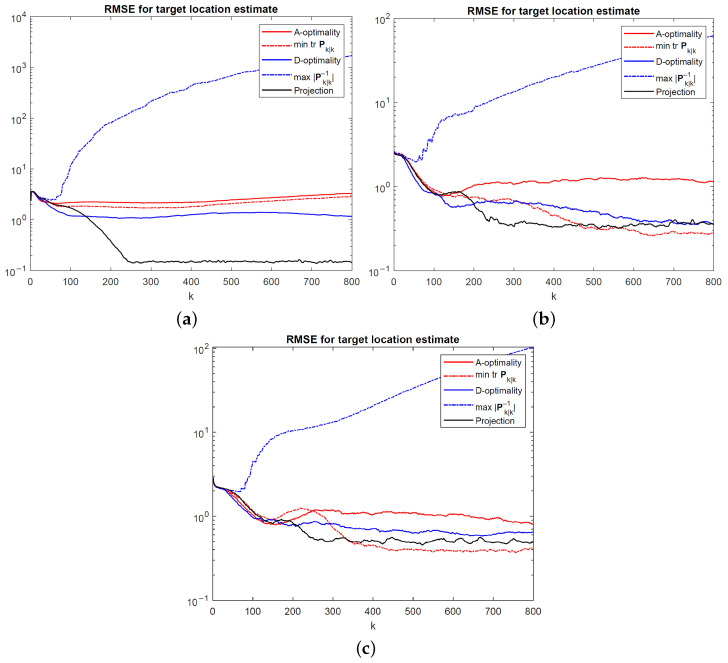
RMSE for EKF target location estimates for a stationary target at (**a**) $\sigma =0.1^{\circ }$, (**b**) $\sigma =1^{\circ }$, and (**c**) $\sigma =2^{\circ }$.

**Figure 12:**
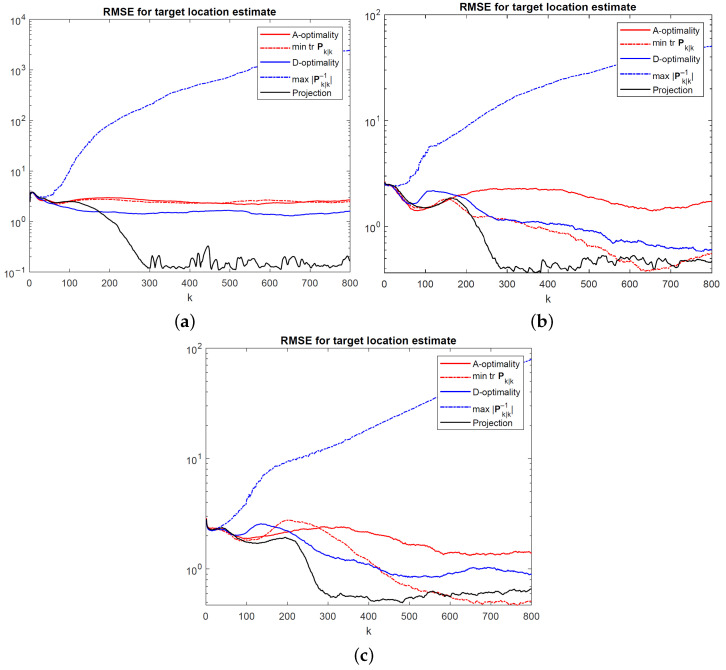
RMSEfor EKF target location estimates for a manoeuvring target at (**a**) $\sigma =0.1^{\circ }$, (**b**) $\sigma =1^{\circ }$, and (**c**) $\sigma =2^{\circ }$.

Figure 11 and Figure 12 show the root mean-square error (RMSE) for EKF target location estimates achieved by the UAV path optimization algorithms for a stationary and manoeuvring target, respectively. The RMSE values were calculated using 400 Monte Carlo simulation runs at the same measurement noise levels as before. In addition to the algorithms developed in this paper, two modifications of the A- and D-optimality-based algorithms are also simulated to illustrate the effects of using the full Kalman filter covariance matrix rather than restricting the optimization problem to the target location covariance. Full Kalman filter covariances were used in [16,21]. In Figure 11 and Figure 12, the modified A-optimality algorithm that minimizes the trace of the filtered state estimate covariance for all state variables (UAV and target kinematic parameters, as well as UAV orientation) is labelled “$\min\mathrm{tr}P_{k|k}$”, and the modified D-optimality algorithm that maximizes the determinant of the inverse covariance matrix for all state variables is labelled “$\max|P_{k|k}^{-1}|$”.

Overall, the projection algorithm achieves the best target tracking performance. At small noise levels, corresponding to high-precision angle measurements, all BFIM-based algorithms perform badly. The modified D-optimality algorithm is observed to have by far the worst performance. This can be explained by noting that the determinant of the full BFIM, which is approximated by $P_{k|k}^{-1}$ in (33), is maximized if $∥b_{i}-s_{k}∥=0$ for any i∈{1,…,N} [see (19)–(21)]. This simply means that the UAV will be attracted towards a nearby beacon and become stuck there, leading to an extremely poor tracking geometry, as evident from the RMSE curves for “$\max|P_{k|k}^{-1}|$” in Figure 11 and Figure 12. Therefore, care should be exercised in particular when using the D-optimality criterion by focusing on the relevant entries of $P_{k|k}$ rather than the whole $P_{k|k}$, as carried out in Section 4.3, to avoid undesirable consequences.

The average RMSE values for the path optimization algorithms for stationary and manoeuvring target cases are listed in Table 1 and Table 2, respectively. The average RMSE is computed by finding the mean RMSE over a time interval during which the RMSE of the projection algorithm has approximately levelled off. Evidently, the projection algorithm achieves the best RMSE performance with the fastest convergence to the minimum RMSE in almost all the simulations.

It is expected that an increased number of beacons will improve not only the UAV self-localization performance, but also the target tracking performance by increasing the eigenvalues of $D_{k}$ [see (37)]. Table 3 and Table 4 show the average RMSE values after increasing the number of beacons to six (N=6) by introducing two more beacons at $b_{5}={\left[22.5,-22.5\right]}^{T}$ km and $b_{6}={\left[-22.5,22.5\right]}^{T}$ km. A comparison with Table 1 and Table 2 confirms that the performance of the projection algorithm and, to a lesser degree, the D-optimality algorithm improves with an increase in the number of beacons. The computational complexity of the EKF is on the order of the cube of the state vector size [28], which is O(729) for the augmented state-space model. Therefore, increasing the number of beacons will not add to the computational complexity significantly.

In general, how well the individual path optimization algorithms perform will depend on the relative beacon–UAV–target geometry, as well as the measurement noise. As is clear from Figure 11 and Figure 12, the achievable RMSE performance in the steady state is proportional to angle noise variances. It is conceivable that as the target moves away from the beacons, the target tracking performance will deteriorate as a result of optimal UAV location being far from the beacons and/or the target, hampering the tracking performance.

## 6. Conclusions

In this paper, we have developed new UAV path optimization algorithms for joint angle-only self-localization and target tracking in the absence of orientation angle information. The recursive BFIM was utilized to derive A- and D-optimality criterion-based algorithms. The potential shortcomings of BFIM-based optimization were considered and demonstrated in simulation studies. The modified projection algorithm, which aims to move the UAV closer to an optimal geometry, was shown to perform well. The developed algorithms are based on a sound foundation in recursive Bayesian estimation theory and make full use of prior information and measurements in relation to target tracking.

UAV self-localization can be further improved by considering a priori knowledge of UAV dynamics, such as the constant velocity and maximum turnrate, optimal beacon placement and dynamic beacon selection as the UAV moves. The self-localization performance will degrade as the UAV moves away from the beacons, producing poor target tracking results. In addition, undesirable self-localization geometries such as near cocircularity of the UAV and beacons will hamper the tracking performance significantly. These situations can be avoided by optimal dynamic beacon selection. An improved integration of self-localization from beacon bearings with an inertial navigation system (INS) onboard the UAV [29] will also enhance the self-localization performance.

## Figures and Tables

**Table 1:** Average RMSE for a stationary target (N=4).

Algorithm	$\sigma =0.1^{\circ }$	$\sigma =1^{\circ }$	$\sigma =2^{\circ }$
A-optimality	2.61	1.19	1.04
D-optimality	1.26	0.49	0.68
Projection	0.15	0.35	0.50
Modified A-optimality ($\min\mathrm{tr}P_{k|k}$)	2.22	0.41	0.43
Modified D-optimality ($\max|P_{k|k}^{-1}|$)	988.6	33.3	39.8

**Table 2:** Average RMSE for a manoeuvring target (N=4).

Algorithm	$\sigma =0.1^{\circ }$	$\sigma =1^{\circ }$	$\sigma =2^{\circ }$
A-optimality	2.42	1.81	1.63
D-optimality	1.56	0.89	0.99
Projection	0.16	0.44	0.59
Modified A-optimality ($\min\mathrm{tr}P_{k|k}$)	2.49	0.65	0.79
Modified D-optimality ($\max|P_{k|k}^{-1}|$)	967	31.1	37.3

**Table 3:** Average RMSE for a stationary target (N=6).

Algorithm	$\sigma =0.1^{\circ }$	$\sigma =1^{\circ }$	$\sigma =2^{\circ }$
A-optimality	2.26	1.25	1.08
D-optimality	1.06	0.38	0.48
Projection	0.05	0.23	0.37

**Table 4:** Average RMSE for a manoeuvring target (N=6).

Algorithm	$\sigma =0.1^{\circ }$	$\sigma =1^{\circ }$	$\sigma =2^{\circ }$
A-optimality	2.45	1.84	1.82
D-optimality	1.30	0.93	0.91
Projection	0.09	0.30	0.51

## Data Availability

Data are contained within the article.
